# Synthesis and Application of Aurophilic Poly(Cysteine) and Poly(Cysteine)-Containing Copolymers

**DOI:** 10.3390/polym9100500

**Published:** 2017-10-11

**Authors:** David Ulkoski, Carmen Scholz

**Affiliations:** Department of Chemistry, University of Alabama, 301 Sparkman Dr., Huntsville, AL 35899, USA; dju0001@uah.edu

**Keywords:** aurophilic, poly(amino acid)s, poly(l-cysteine), drug delivery, surface modification, electrochemical based detection

## Abstract

The redox capacity, as well as the aurophilicity of the terminal thiol side groups, in poly(Cysteine) lend a unique characteristic to this poly(amino acid) or polypeptide. There are two major application fields for this polymer: (i) biomedical applications in drug delivery and surface modification of biomedical devices and (ii) as coating for electrodes to enhance their electrochemical sensitivity. The intended application determines the synthetic route for p(Cysteine). Polymers to be used in biomedical applications are typically polymerized from the cysteine *N*-carboxyanhydride by a ring-opening polymerization, where the thiol group needs to be protected during the polymerization. Advances in this methodology have led to conditions under which the polymerization progresses as living polymerization, which allows for a strict control of the molecular architecture, molecular weight and polydispersity and the formation of block copolymers, which eventually could display polyphilic properties. Poly(Cysteine) used as electrode coating is typically polymerized onto the electrode by cyclic voltammetry, which actually produces a continuous, pinhole-free film on the electrode via the formation of covalent bonds between the amino group of Cysteine and the carbon of the electrode. This resulting coating is chemically very different from the well-defined poly(Cysteine) obtained by ring-opening polymerizations. Based on the structure of cysteine a significant degree of cross-linking within the coating deposited by cyclic voltammetry can be assumed. This manuscript provides a detailed discussion of the ring-opening polymerization of cysteine, a brief consideration of the role of glutathione, a key cysteine-containing tripeptide, and examples for the utilization of poly(Cysteine) and poly(Cysteine)-containing copolymers, in both, the biomedical as well as electrochemical realm.

## 1. Introduction

Physiologically, poly(l-Cysteine), p(l-Cys), does not exist as a homopolymer and there are no known extended p(l-Cys) sequences neither in normal nor pathologic proteins, as there are e.g., extended sequences of p(l-Glutamine) associated with Huntington’s disease [1,2] and poly(l-Alanine) sequences indicated in oculopharyngeal muscular dystrophy [3]. In synthetic polymers, l-Cys building blocks have been utilized in combination with other amino acid building blocks primarily for biomedical applications and a chemically different form of p(Cys) has been exploited in electrochemical settings for the detection of chemical substances. This article discusses briefly the role of l-Cys building blocks in proteins and peptides from a polymer science point of view. 

The main focus of the article will be on the synthesis of p(Cys) and p(Cys)-containing copolymers and will highlight the two main—but very different—applications for the polymers: (i) biomedical polymers for drug delivery and surface modification of biomedical devices exploiting p(Cys)’s redox sensitivity and aurophilicity and (ii) electrochemical-based detection of chemical moieties. The change in the physiochemical state of l-Cys within different physiological settings (either acidic or basic) is due to its redox potential. Therefore for drug delivery, cysteine-containing polymers have the potential to provide controlled structural manipulation or disassembly in different intracellular environments, creating a delivery vehicle capable of releasing a drug within the cytoplasm of cells. It should be noted that aurophilicity of polymers should be understood as the reactivity of a polymer towards gold moieties, typically the formation of a bond between a thiol group and a gold surface or particle. This is in contrast to the inorganic definition of aurophilicity that refers to the formation of weak gold-gold bonds in the aggregation of gold complexes [4].

Throughout the article, the abbreviations l-Cys and p(l-Cys) will be used for l-Cysteine and poly(l-Cysteine) if the stereochemistry is known. If this is not the case, the generic forms Cys and p(Cys) abbreviations will be used. In the biological domain, only the l-enantiomer exists. Furthermore, polymers consisting of amino acid building blocks are referred to as polypeptides or poly(amino acid)s, PAAs. Throughout this manuscript the term PAAs will be preferred.

## 2. The Role of l-Cysteine Building Blocks in Proteins and Peptides

Biochemically, l-Cys is formed either from l-Methionine or as a product of the sulfate assimilation cycle. In proteins, the l-Cys residue fulfills a stabilizing role, largely by ‘locking in’ the protein fold structures through disulfide bridges with other l-Cys residues, thereby oxidizing l-Cysteine to l-Cystine. These disulfide bridges form within a protein and stabilize the respective protein fold structure, that is the protein’s tertiary structure, or they form between different protein chains, which lead to the formation of quarternary protein structures. The disulfide bridges in proteins are unstable in reducing environments such as the cytosol. In addition to the disulfide bridges, l-Cys residues further contribute to stabilizing proteins through their hydrophobic interactions. While a thiol and a hydroxyl-group are chemically rather similar, the chemical behavior of l-Cys and l-Serine in proteins is rather different; l-Cys is found more often in the hydrophobic regions of proteins. While there are some discrepancy among various hydrophobicity scales for amino acids [5,6,7,8], the consensus dictates that l-Cys is considered to be polar and hydrophobic and stabilizes proteins through hydrophobic interactions. The hydrophobic character of synthetic p(l-Cys) also reveals itself in its biologic interactions. As indicated before, there is no known naturally occurring p(Cys), however a study of the cellular effects of artificially produced p(l-Cys) showed that it behaved like a hydrophobic homopolymeric amino acid sequence. That is, it upregulated enzyme activity that is associated with dying cells [9]. The study indicated that the cytotoxicity of p(Cys) is on par with poly(l-Isoleucine), poly(l-Leucine) and poly(l-Valine). There is a correlation between a homopolymeric amino acid sequence and its cytotoxicity and p(l-Cys) clearly ranged on the hydrophobic side. 

**Scheme 1:**
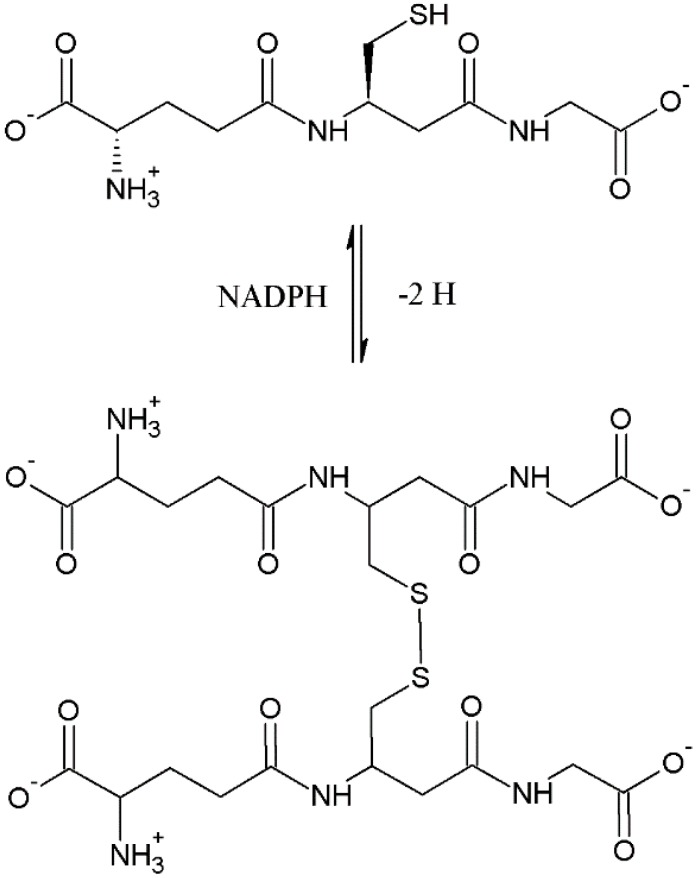
The redox equilibrium of reduced glutathione and oxidative formation of glutathione disulfide.

While there are no known physiological p(l-Cys) structures, some attention should be directed towards the physiological role that l-Cys plays as a single amino acid within peptides. l-Cys holds a vital position in the sulfur metabolism and fulfills a unique role in cellular redox reactions. As most chemical reactions involving l-Cys are redox reactions, the reversibility of these reactions make l-Cys a versatile chemical transducing element that is indispensable in low molecular mass metabolite formation as well as in structural biology [10]. Most notably, and most thoroughly investigated is the formation of glutathione, Scheme 1, a potent antioxidant that counteracts various reactive oxygen species, thereby preventing damage to cellular components. Gluthathione forms when free l-Cys is produced from cystathionine through the action of Cystathionine-γ-lyase, an enzyme that catalyzes the cleavage of cystathionine to l-Cys, which is then ligated with l-Glutamate, catalyzed by γ-glutamylcysteine synthetase. Glutathione is eventually formed by adding glycine to the C-terminal of γ-glutamylcysteine, catalyzed by glutathione synthetase [11]. Glutathione evolved as suitable cellular thiol redox buffer because it is not as toxic as the free amino acid l-Cys [12]; as such it maintains cellular thiol/disulfide redox potentials. More recently glutathione has been implicated in a plethora of biological processes ranging from gene expression, apoptosis and signalling functions to implications in cellular and nerve functions [13,14,15]. A number of excellent reviews on glutathione are available [11,16,17,18]. 

Chemically, glutathione serves as an electron donor and gets oxidized to glutathione disulfide. It protects cells from oxidative stress by donating a reducing equivalent to reactive oxygen species, thereby becoming oxidized and forming a disulfide linkage to another oxidized glutathione. It can be reduced back to glutathione with nicotinamide adenine dinucleotide phosphate (NADPH), serving as an electron donor. The ratio of reduced to oxidized glutathione serves as an indicator for cellular oxidative stress. The antioxidant function of glutathione is not only utilized by mammals, including humans, but has also been observed in plants, fungi and some bacteria. 

## 3. Poly(Cysteine) Synthesis

### 3.1. Historical Overview

Attempts to synthesize p(Cys) go as far back as 1951 when Jones and Lundgren attempted to synthesize p(Cys) in an effort to better understand the mechanical properties of wool [19]. In that study, Cys *N*-carboxyanhydride (Cys-NCA) was prepared from *N*,*N*′-dicarboethoxy- l-cystinylchloride, following the method originally described by Leuchs in his trailblazing 1906 publication [20] and the polymerization was initiated by heating either the *N*,*N*′-dicarboethoxy- l-cystinylchloride or the l-Cys-NCA that results from it upon heating in a variety of “moist solvents”. They reported that the resulting product was insoluble in most common solvents, most likely the product heavily crosslinked due to disulfide formation, however, the product was soluble in mineral acids and alkalies, most likely due to hydrolysis rather than dissolution. Further efforts by Sakakibara and Tani to produce p(Cys) focused on investigating the unique role that Cys residues have in natural proteins. They seem to be the first researchers who employed phosgene for the NCA formation, they also protected the thiol side group as a disulfide, and polymerized the S-thiophenyl protected l-Cys in ethyl thioglycolate, which brought about the polymerization and simultaneously the reduction of the *S*-thiophenyl protection group to yield p(Cys) [21]. They counteracted the disulfide formation by exposing the polymerization product to tin and HCl or by exposing it to excess mercaptane. 

### 3.2. Synthesis of Poly(Cysteine) and Other Poly(Amino Acid)s by Controlled Ring Opening Polymerization of Amino Acid N-Carboxyanhydrides

Significant progress in the field of controlled ring-opening polymerization (ROP) of amino acid NCAs was made by Deming who first reported on a well-controlled NCA ROP employing metal catalysts and thereby establishing living polymerization conditions. Given the significant advantages of NCA ROP, this is the most common synthetic technique to produce p(l-Cys) and poly amino acids (PAAs) in general for biomedical applications. The advantage most valuable to biomedical polymers is the ability to control the molecular weight and guarantee a very narrow PDI. Deming’s zero-valent nickel complex initiator (2,2′bipyridyl-Ni-cyclooctadiene) provides an active site for the amino acid NCA but is less susceptive to side reaction due to steric hindrance and for electronic reasons [22,23,24]. Later Hadjichristidis introduced a metal-free high vacuum technique, which ensures ultrapurity of all reagents throughout the entire synthesis to produce PAAs [25]. This technique is advantageous as it does not employ any metal initiators, which is of special interest for polymers intended for biomedical applications, however it is laborious as extensive care needs to be taken to render the apparatus absolutely water-free. Just like Deming’s method, the polymerization follows a living mechanism, and guarantees control over molecular weight and composition. Primary amines are used as initiator and the polymerization follows the regular amine mechanism as introduced by Kricheldorf [26]. Schlaad used amine salt macroinitiators for NCA polymerizations at elevated temperatures, which can eliminate the formation of unwanted NCA anions and lower the polydispersity [27]. Increased reaction temperatures (40–80 °C) are needed to increase the equilibrium concentration of free amine and produce a higher reaction rate. However, these authors never synthesized p(l-Cys) using this method. Schue et al. determined that PAAs could be produced in a controlled manner from amino acid NCA in a living polymerization and absent of the extreme high vacuum conditions developed by Hadjichristidis when the polymerization kinetics were slowed down by conducting the polymerization at 0 °C [28]. 

### 3.3. The Role of Urea in the Synthesis of Poly(Amino Acid)s

We expanded on Schue’s technique by identifying the strong tendency to form secondary structures in nascent PAA chains, which leads to a hindrance in the progression of a controlled ROP of amino acid NCAs and loss of the living character of the polymerization. As long as the growing chains are too short to turn into an α-helix they are prone to interact with one another via hydrogen bonds. These hydrogen bonds cause the formation of β-sheets [29], which are rigid and suppresses the ability of the growing chain to undergo continued nucleophilic attacks on NCA monomers and tend to precipitate from the polymerization, effectively terminating the growth of the affected chains. Typically growing PAA chains adopt helical conformation upon reaching a length of 10 to 20 repeat units, but l-Cys has a very strong tendency to form β-sheets and will not adopt a helical conformation at any chain length, which makes the formation of p(Cys) from l-Cys NCA particularly demanding [30]. 

However, hydrogen-bonds between growing polymer chains can be broken, e.g., by small molecules such as urea, thiourea or guanidine. Conducting the polymerization of amino acid NCAs in the presence of urea enables a living polymerization following the regular amine mechanism as the formation of secondary structures during the early stages of the polymerization is successfully suppressed and moreover, polymerizations can be conducted at room temperature while maintaining a living character. It is of importance that the use of metal catalysts is avoided, as is the use of an instrumentally challenging high vacuum apparatus [31,32,33]. 

### 3.4. General Synthetic Approach for the Synthesis of Poly(l-Cysteine) at Room Temperature and in the Presence of Urea

**Scheme 2:**
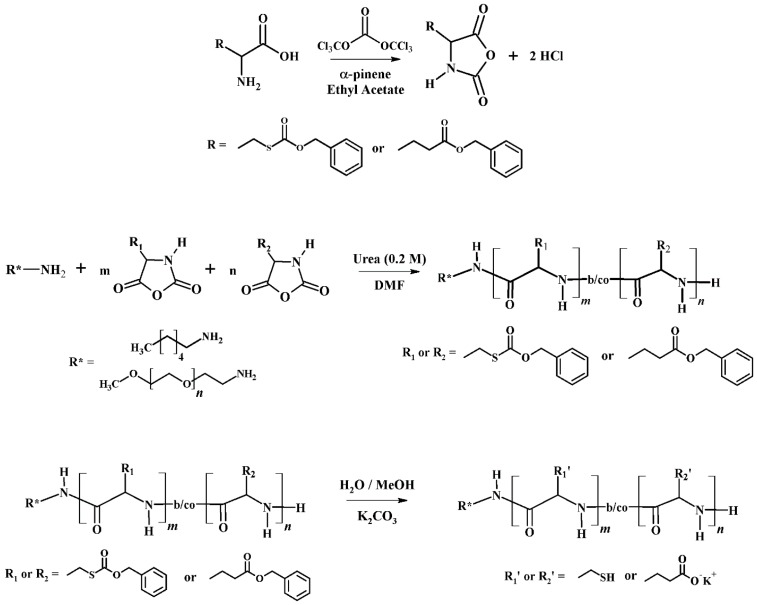
Synthetic process for production of PEG-b-p(l-Cys)_x_-b-p(l-Glu)_y_ and PEG-b-p((l-Cys)_x_-*co*-(l-Glu)_y_) block and random copolymers through amine-initiated ring-opening polymerization of amino acid *N*-carboxyanhydrides (NCAs).

The following general approach has proven successful for producing PAAs including p(Cys) and p(Cys)-containing copolymers—see Scheme 2. First, NCA is synthesized in a reaction of the amino acid with a phosgene derivative, e.g., triphosgene. It should be noted that NCA monomers have multiple reaction sites: two carbonyl groups and nucleophilic positions consisting of an amine and a methine. These reactive regions can lead to the formation of multiple different side-products and need to being taken into account when selecting the proper initiator [26]. It is essential that ROPs are carried out under inert, anhydrous conditions, hence Schlenk lines are most suited for these polymerizations. The NCA monomer and the urea must be dry prior to the polymerization. The solvent, typically dimethylformamide (DMF), must be freshly distilled and is added to the urea, typically to achieve approximately a 0.1 M concentration, the mixture should be thoroughly degassed at this point. This DMF/urea solution is then added to the NCA, under a flow of argon. An aliquot of an initiator stock solution is quickly added to the NCA/urea/solvent mixture to initiate the polymerization under a flow of argon. The polymerization proceeds at room temperature and as the polymerization progresses, CO_2_ is produced. The CO_2_ production serves as indicator for the progress of the polymerization; it is monitored most readily via the back pressure in the Schlenk line. Once re-purging the Schlenk line with argon, it is recommended to check the CO_2_ production. The reaction is complete when the gas evolution has ceased.

Once the polymerization step is complete, the polymer reaction solution is concentrated in vacuo and urea is removed through its recrystallization in a non-solvent like tetrahydrofuran (THF). After removing the urea precipitate (ensuring that only urea was precipitated by dissolving it in water is recommendable), the filtrate is concentrated again and dialyzed against deionized water for several days with multiple water replacements. Dialysis has been found to be the superior purification method because it allows for the removal of any remaining urea, DMF, or THF. Poly(l-Cys) and other PAAs with functional side groups that are protected during the polymerization can now be deprotected. Several deprotection reactions are available and chosen mainly based upon the chemical nature of the amino acid. Poly(l-Cys) is typically protected as carboxybenzyl group and deprotects readily under basic conditions [34]. Methanol/water with potassium carbonate was found to be very effective. The polymer is recovered by lyophilization.

The living nature of ROP is exploited when PAA block copolymers are synthesized. The polymerization of the NCA of the first amino acid is initiated and after CO_2_ production from this polymerization ceases, the NCA of the second amino acid can be added. Since the polymerization is living, the amino-terminated PAA chain will reinitiate the polymerization of the second amino acid NCA. This polymerization will again be monitored via CO_2_ production until the gas evolution as indicated by a pressure increase stopped, indicating that the second monomer is completely consumed as well. PAA copolymers are readily synthesized in one step by adding NCAs of the different amino acids simultaneously to the initiator. Amino-terminated macroinitiators, such as α-methoxy-ω-amino poly(ethylene glycol) (PEG) and α-hydroxy-ω-amino-poly(2-methyl-2-oxazoline) as well as small molecule amines have been shown to be effective in initiating NCA ROP as described above [29,32,33,35].

## 4. Poly(Cysteine) Applications

### 4.1. Poly(Cysteine) in Biomedical Applications

PAAs have gained substantial interest as biocompatible polymers due to the fact that (i) their degradation products are biocompatible l-amino acids and (ii) the absolute isotacticity of the polymers, which results from their enantiomerically pure l-amino acid building blocks, allows for the formation of secondary structures that contribute further to the overall biocompatibility of these biopolymers. These stable secondary structures that PAAs adopt in aqueous solution lend them a superior behavior over conventional synthetic polymers and promotes self-assembly that can be exploited in biomedical applications, such as drug delivery, tissue engineering, and polymer coatings [26,36,37,38]. Poly(l-Cysteine), the polymer of interest in this article forms preferably β-sheets [26]. Aside from the secondary structure, the amphiphilicity and reactivity of PAAs imparted by their side chains determine their physical properties and subsequent utilization in biomedicine. The redox-active thiol group in p(l-Cys) oxidizes readily thereby cross-linking the polymer. 

The biocompatibility of PAAs can be enhanced by forming block copolymers with other biocompatible polymers such as PEG and poly(oxazoline). PEG in particular holds a special place among biocompatible polymers. It is a polyether that is a widely used as biomedical polymer, as it is capable to induce a stealth character upon PEGylated moieties and surfaces [39,40]. PEG-PAA copolymers were shown to exhibit low toxicity and high hydrophilicity [41,42], which makes them acceptable for therapeutic applications, such as drug and gene delivery [43]. PEG has excellent blood compatibility and its stealth character provides the ability to repel proteins [44,45]. In aqueous medium, PEG exhibits rapid chain motion and a large excluded volume resulting from low interfacial free energy and loss of configurational entropy of PEG chains in the presence of a foreign particle, respectively [46,47,48,49]. Hence, PEGylated biomaterials are biocompatible and possess increased stability in nanosystems, while reducing thrombogenic and antigenic effects of potential macromolecules and specific antibodies. PEG has therefore often been used as a hydrophilic block in block copolymers with various PAAs. 

### 4.2. Poly(l-Cysteine) in Surface Modifications

The surface of any implanted biomedical device that interfaces with the tissue is chiefly responsible for the success or failure of the device. While the nature of the bulk material of the device is determined by stability, flexibility, electrical and other parameters, it is typically not very biocompatible and modifications of device surfaces are desirable [50,51].

**Figure 1:**
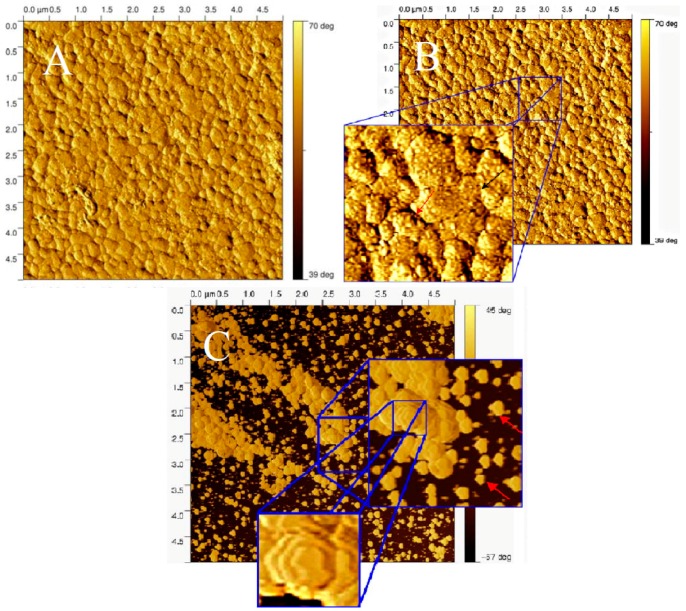
AFM phase images of PEGylated poly(amino acid) coatings on the gold on silica wafer. PEG-b-p(l-Glu)_100_-b-(l-Cys)_20_ (**A**,**B**) are shown on gold surface; while PEG-b-p((l-Glu)_50_-*co*-(l-Cys)_10_) (**C**) is coated onto large gold nanoparticles on silicon surfaces. Figure 1B shows nodules of PAA phase separation. Source: Obeid 2014 [37]. Reproduced with permission from Wiley Periodicals, Inc.

The aurophilicity of the thiol group of p(l-Cys) can be utilized wherever a device is manufactured from or encased in gold. As shown in Figure 1A, a complete coverage of a gold surface was achieved with PEG-b-p(l-Glu)_100_-b-p(l-Cys)_20_ showing the typical “orange peel” morphology. This “orange peel” morphology is due to the heterogeneity of the polymer layer that results from the phase separation of the mutually immiscible PEG and PAA blocks. Changing from a PAA block copolymer to random copolymer (PEG-b-p(l-Glu)_100_-*co*-p(l-Cys)_20_), did not impact the overall surface morphology. However, at higher magnification, Figure 1B, small spherical domains, a few nanometers in diameter, become visible for the PAA block copolymers that are absent in PAA random copolymers. It was assumed that this additional phase separation resulted from the phase separation of the two PAA blocks. In general, the patterns are consistent and independent of the molecular weight of the respective PAA segments. The key feature for the modification of surfaces with p(l-Cys)-containing copolymers are multiple l-Cys repeat units per chain that dictate a multi-anchor attachment of the polymer [38,52], which ensures the stability of the coating.

The aurophilicity can be equally exploited for the coating of gold nanoparticles deposited on a surface. The coating is highly specific to the gold nanoparticles and the intermittent silica surface is not coated when the nanoparticles are well defined and comparatively far apart from one another; Figure 1C shows the coating of 200–400 nm gold nanoparticles with PEG-b-p((l-Glu)_50_-*co*-(l-Cys)_10_). The coating of gold nanoparticles with PEGylated p(l-Cys) containing copolymers allows for some insight into the coating mechanism. As the p(l-Cys) repeat units of the PAA block are covalently bound to the gold nanoparticles, the PEG block must reside on the top of the polymer layer. The observed morphology, Figure 1C clearly results from the crystallization of the PEG block and resembles screw dislocations [53]. When the nanoparticles are close together the polymer bridges several nanoparticles, therefore, producing a continuous coating. This is of particular interest for the coating of devices that cannot be encased in solid gold, for example, for electrical reasons, but a rather uninterrupted coating can be achieved on top of gold nanoparticles. By exposing the polymer coating to an aqueous environment, as it is the case at the device–tissue interface, the PEG blocks will protrude into the water and configure into brush-like polymers that then generate the stealth-like surface characteristics.

### 4.3. Poly(l-Cysteine) in Drug Delivery Vehicles

**Scheme 3:**
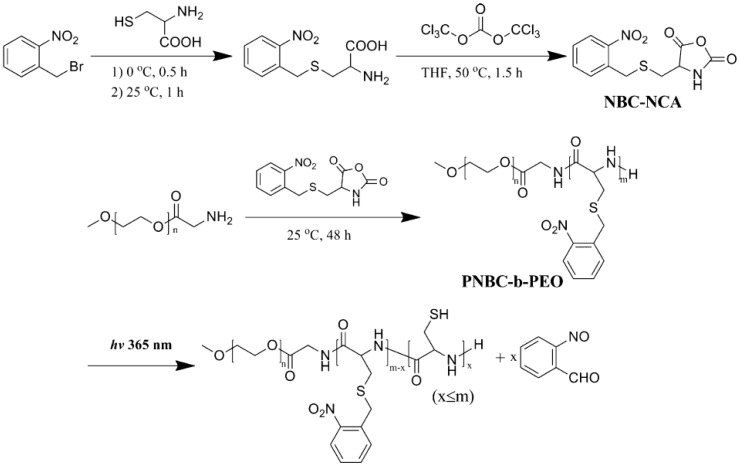
Synthesis of Photoresponsive *o*-nitrobenzyl-l-Cys NCA Monomer and the subsequent synthesis and photocleavage of PEGylated *o*-nitrobenzyl-derivatized p(l-Cys) Block Copolymers. Source: Wu et al. 2016 [54]. Reproduced with permission of American Chemical Society.

An elegant and elaborate dual drug delivery system that combined drug delivery with photothermal therapy based on p(l-Cys) was developed by Dong et al. The authors developed a chemistry based on the photoresponsiveness of *o*-nitrobenzyl-protected l-Cys building blocks. The *o*-nitrobenzyl-protected Cys NCA was polymerized by ROP using an amino initiator, e.g., α-methoxy-ω-amino PEG. This construct in itself can be used as drug delivery system, as the block copolymer is amphiphilic and self-assembles into nanoparticles in aqueous solutions and drugs can be incorporated during the self-assembly step. The *o*-nitrobenzyl-derivatized p(l-Cys) was found to have a thermotropic liquid crystal phase behavior, which supported the formation of stable p(*o*-nitrobenzyl-l-Cys) β-sheets as the nanoparticle core. As seen in Scheme 3, UV-Irradiation of this block copolymer leads to the photo-cleavage of the *o*-nitrobenzyl group and therefore release of a physically entrapped drug [54]. The authors expanded the concept, in particular by exploiting the slow and therefore controllable photocleavage of the *o*-nitrobenzyl protective groups. Stopping the photodegradation at a pre-determined level of deprotection yields a certain amount of free thiol groups that can be derivatized by thiol-ene conversions with e.g., acrylate moieties. Using PEG acrylate yields a comb-like copolymer with a p(l-Cys) backbone, grafted with PEG and the degree of grafting is controlled by the degree of photodegradation of the *o*-nitrobenzyl groups. These comb-like copolymers self-assemble into vesicles or micelles depending on grafting ratio; with copolymers with lower grafting ratios (less than 30%) forming vesicles; at very high grafting ratios (above 75%) no self-assembly was achieved [55,56]. This is an interesting observation as the self-assembly of PEGylated polyaminoacids/polypeptides into micelles and nanoparticles or aggregates is often described, however, vesicles are rarely observed [57]. Further irradiating these comb-like copolymers led to further deprotection and the formation of additional free thiol groups. These newly formed thiol groups were oxidized with hydrogen peroxide, which led to cross-linking via disulfide linkages and the formation of stable nanoparticles. Doxorubicin was incorporated into nanoparticles and a 100% drug release was achieved within 60 h when UV irradiation (3 × 3 min) and dithiothreitol (DDT), which mimicked glutathione present in the body, were used as drug release triggers.

**Scheme 4:**
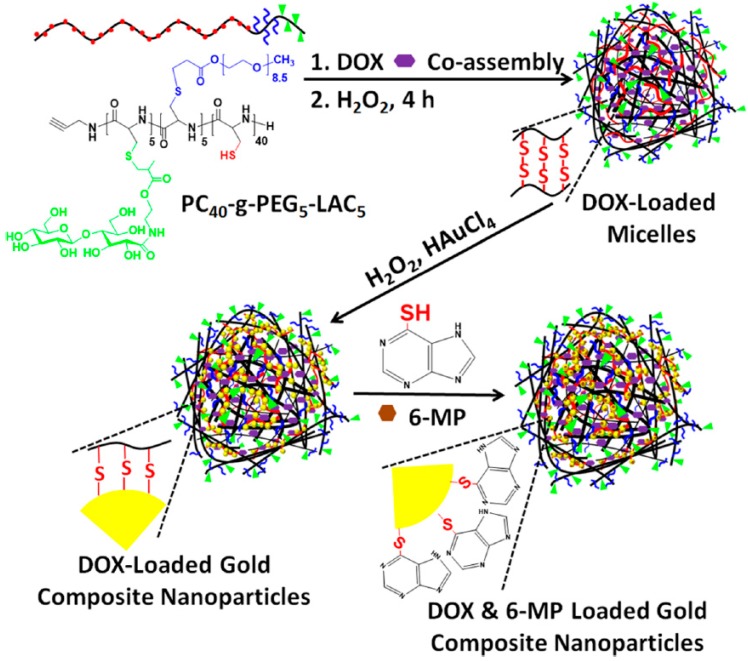
Assembly of doxorubicin (DOX) and 6-mercaptopurine (6-MP) dually loaded polypeptide/gold composite nanoparticles. Source: Wu et al. 2016 [58]. Reproduced with permission of American Chemical Society.

Dong et al. further expanded on the concept by exploiting the inherent aurophilicity of Cys and by adding lactose as a homing device to the copolymeric construct, Scheme 4 [58]. Copolymers were synthesized as described above, and the *o*-nitrobenzyl protective groups were removed sequentially, allowing for the subsequent modification with PEG and lactose. Further irradiation of this graft copolymer resulted in free thiol groups which oxidized, when nanoparticles were formed. Exposing these nanoparticles to tetrachloroauric acid in the presence of hydrogen peroxide, which acts as a mild reductant in the presence of auric acid, led to the formation of gold nanoparticles, to which the free thiol groups covalently attach. Moreover, the presence of the gold nanostructures within the polymeric nanoparticle allows for loading the structure with a second, thiol-containing drug, that binds to the gold structures. Thus, a PEGylated polypeptide equipped with a lactose homing device was constructed, self-assembled and stabilized through gold nanostructures that were formed in situ and loaded with two different drugs. This construct enables a synergistic chemo- and phototherapeutic approach, as the gold containing particles showed strong surface plasmon resonance absorption in the VIS-NIR spectrum. 

**Scheme 5:**
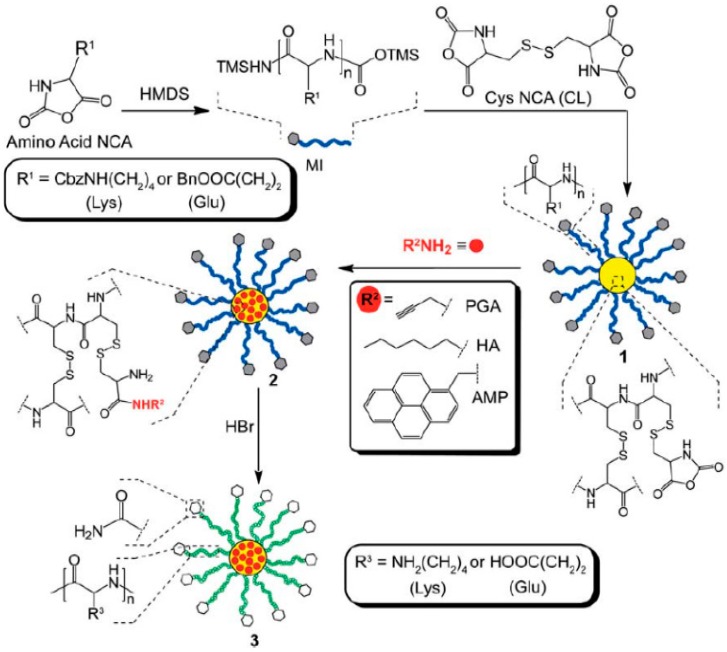
Synthesis of amino acid-based polymers containing controlled functionalities via a one-pot, arm-first strategy. Source: Sulistio et al. 2012 [59]. Reproduced with permission of Royal Society of Chemistry.

Qiao et al. prepared star polymers by exploiting the cross-linking capacity of p(l-Cys—which served as the core of the star polymer and another PAA—p(l-Glu) or p(l-Lys) formed the arms, Scheme 5 [59]. The star polymers were produced in a one-pot synthesis, where the ‘arms’ of the star were polymerized first by the ROP of l-Lys or l-Glu NCA. These authors used a secondary amine, hexamethyldisilazane, as initiator, which they found to provide better control over the polydispersity than the typically used amino initiators. Once the linear arms were formed the NCA of the cystine dimer was added, that is, two NCA functionalities per monomer, and polymerized. This polymerization yielded a cross-linked structure due to the bifunctionalilty of the cystine monomer. Star formation depended largely on an optimum cross-linker (cysteine) to macroinitiator (‘arm’ of the star) ratio, which by itself heavily depended on the molecular weight of the macroinitiator [60]. Since not all NCA units on the cystine monomer participate in the ROP, the core of the star polymer can be subsequently derivatized by opening these unreacted NCA groups with amines. Deprotecting the ‘arms’ of the star polymer yielded a p(l-Lys) or p(l-Glu) corona for the star polymers, thereby making the construct water soluble. Equally, the ‘arms’ of the star polymers can be derivatized after deprotection. For example, p(l-Glu) was directly converted into a hydrazide derivative by conversion with hydrazine, thereby providing a route for drug conjugation. 

Heise et al. have shown that the ROP of amino acid NCA, as described above in the p(Cys) synthesis section can also be accomplished using silica nanoparticles that were aminated with 3-aminopropyltrimethoxysilane and acted as initiators, in a grafting-from type polymerization. Specifically, p(benzyl-l-Glutamate), p(carbobenzyloxy-l-Lysine) and p(tert-butyl l-Cysteine) were grafted on these silica nanoparticles using a typical NCA ROP. The resulting constructs can be deprotected and derivatized and may find applications based on the unique optical and electrical properties imparted by the silica and the reactivity and responsiveness imparted by the various PAA shell [61].

P(l-Cys) derivatized with short ethylene glycol (diethylene glycol to octaethylene glycol) side chains form mostly β-sheets in aqueous solution, indicating that the natural p(l-Cys) conformation supersedes the solubility introduced by the ethylene glycol moieties [62]. Oxidizing this thioether to either a sulfoxide or sulfone group increases the polarity and most importantly induces a change in the secondary structure from β-sheets to random coils as evidenced by the change from a turbid suspension to a clear solution. The same oxidation-triggered hydrophobic to hydrophilic transition was observed when the ROP of oligo(ethylene glycol) (meth)acrylate l-cysteine NCA was initiated with an amino-terminated PEG, only here the resulting PEG-b-p(l-diethyl methacrylate cysteine) diblock copolymers formed polymeric micelles in aqueous solution. Again, oxidizing the thioether side chains of the derivatized p(l-Cys) to the corresponding sulfoxides or sulfones led to a nearly complete deconstruction of the polymeric micelles [63]. As shown by circular dichroism the thioether form of the diblock copolymer adopted preferably a β-sheet conformation, which is remarkable as PEG with a molecular weight of 2000 Da constitutes one block of this diblock copolymer, yet the natural secondary structure of p(l-Cys) dominates despite the extensive hydrophilic presence of the PEG block and the ethylene glycol side chains. Only the conversion of the thioether into a sulfoxide or even sulfone breaks the β-sheets, leaving the diblock copolymer in a random coil. The authors indicate that this oxidation-triggered disassembly might be utilized for the delivery of anti-inflammatory drugs as inflammatory cells often exhibit an oxidative environment [64]. 

Cai et al. synthesized derivatized p(l-Cys) containing copolymers for applications in tumor-targeted drug delivery systems, which provided a dual, pH and redox, response system [65]. A poly(l-Lysine)-*co*-poly(l-Cysteine) copolymer was produced by regular ROP of the respective benzyloxy-protected NCAs. After deprotection, the p(l-Lys) building blocks of the copolymer were partially derivatized with folic acid, using 1-ethyl-3-[3-(dimethylamino)propyl] carbodiimide/*N*-hydroxysuccinimide (EDC/NHS) coupling, and in a subsequent second derivatization step the remaining amino groups of p(l-Lys) were derivatized with dicarboxylic acid cyclohexene anhydride or succinic anhydride. Upon dissolving these derivatized copolymers in buffer they self-assembled into nanoparticles and the p(l-Cys) moieties cross-linked. Folic acid will act as homing device for these nanoparticles as folic acid receptors are overexpressed in some cancer cells. Folic acid will also generate a positive charge on the nanoparticles. The anhydride moiety provides a negative charge to the nanoparticle at physiological pH, but turns into a positive charge under the weak acidic conditions in the endosomal and lysosomal compartments. Upon exposure to an acidic pH and in the presence of glutathione the nanoparticles disintegrated and released their drug (doxorubicin) cargo. The drug release increased from 40% at pH 7.4 to 70% at pH 5.0, over a 24 h period. Adding DDT, which mimicked glutathione to this in vitro assay, increased drug release to 60% and 95%, respectively. A stronger drug uptake was observed in folic acid positive cancer cells as compared to folic acid negative cancer cell lines.

Other than the obvious candidates for p(Cys) block copolymerization, which are PEG and other poly(amino acid)s, poly(l-lactide) [66] and poly(styrene) [67] have also been used in p(Cys) containing block copolymers. In the latter case, spherical aggregates were formed with a poly(styrene) core and a p(l-Cys) corona; adding gold nanoparticles to these aggregates forced the architecture to reverse itself. Due to the stronger affinity of the thiol groups for the gold poly(styrene) was forced to form the corona around the gold nanoparticle-p(l-Cys) cores. Drug release from shell-cross-linked aggregates formed from poly(l-lactide-b-poly(l-Cys) was shown to be dependent on the redox environment that these drug delivery aggregates were exposed to. The presence of DTT doubled the drug release from 40 to 80% within 5 h in PBS buffer [53].

**Scheme 6:**
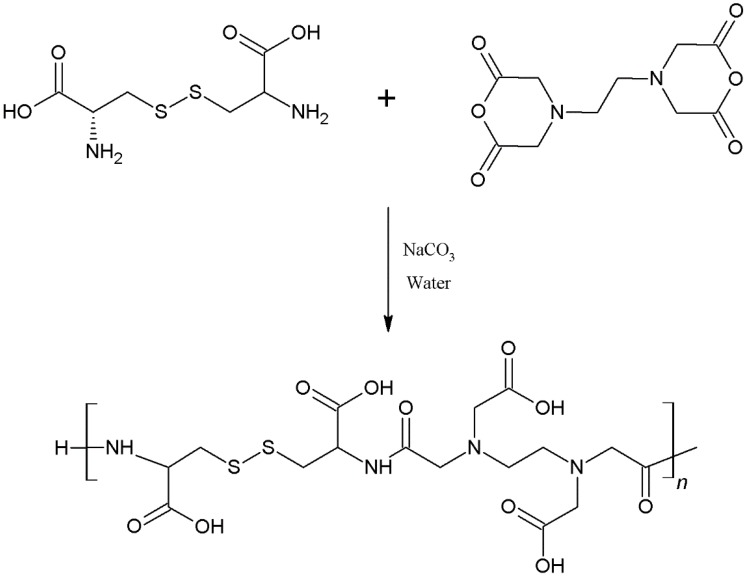
Production of polyurethane containing pH-sensitive disulfide bridge through condensation of l-cystine and EDTA-dianhydride. Source: Sethuraman et al. 2008 [68]. Reproduced with permission of Springer.

Bae et al. took a radically different approach to Cys-containing copolymers [68]. Instead of using the pendant thiol groups for subsequent polymer modification, they used the l-cystine dimer and converted its amine functions with ethylenediaminetetraacetic acid (EDTA) dianhydride. The resulting polyurethane has disulfide linkages in its backbone and four pendant carboxyl groups per repeat unit that can be further derivatized, Scheme 6. After modifying this construct with PEG for biocompatibility and pH-sensitive sulafadiazine pendant groups, which are negatively charged at neutral pH, it served as anchor moiety for a positively charged peptide homing device. In the acidic environment of a tumor cell the pH-sensitive moiety loses its charge and thereby releases the peptide homing device and the drug carrying polymeric micelle it is attached to. Thus, the anticancer drug is delivered directly to cancer cells and pH neutral cells remain unaffected.

### 4.4. The Use of Poly(l-Cysteine) in Electrochemistry as Detection Reagent

Initially, p(Cys) was found to be a potent metal chelator. Holcombe et al. investigated p(Cys) for its ability to interact and chelate metal ions and separate various metals [69,70,71,72,73,74,75]. Commercially available, short p(Cys) homopolymers with about 20 repeat units were covalently attached to substrates that could be rendered to expose carboxylic acid groups on their surface. After activating these carboxylic acid groups p(Cys) was coupled covalently to the substrate via its amino terminus using an EDC coupling technique. It was determined that p(Cys) is better suited in chelating metals than surface bound l-Cys [76]. In more recent studies p(Cys) was reported in a variety of electrochemical settings, specifically for the detection of a multitude of chemically, and biologically relevant compounds. In those studies, it is reported that p(Cys) films were deposited onto electrodes, primarily glassy carbon electrodes, which led to improved electrochemical performances of these electrodes indicated by larger peak currents as compared to their uncoated counterparts. The improved performance was attributed to the p(Cys) microstructure [77,78,79,80,81,82,83,84]. The enhanced electrochemical sensitivity allowed for the detection of individual components in mixtures with a signal resolution that could not be achieved with regular bare glassy carbon electrodes [85,86]. An even further enhancement in signal resolution was achieved by incorporating metals into the p(Cys) films. Gold nanostructures such as nanorods can be readily incorporated by covalent attachment to the p(Cys) films [87,88]. A similar sensitivity enhancement was achieved by doping the p(Cys) layer with silver [82,89,90].

However, a closer inspection of these reports reveals that p(Cys) films for electrochemical applications are deposited by cyclic voltammetry, also referred to as electrochemical or anodic deposition, or electro-polymerization. The electrode material, typically glassy carbon, is immersed in a diluted cysteine solution, typically in buffer and exposed to cyclic voltammetry between −0.6 and 2.0 V, most often 20 cycles are performed. During cyclic voltammetry Cys is electro-oxidized and the resulting amine cation radical reacts with edge plane sites of the aromatic carbon moiety that forms the electrode material thereby forming a nitrogen-carbon bond, hence, the preference for glassy carbon electrodes. The electrochemical oxidation of amine-containing compounds, that is, the formation of the amino cation radical has been elucidated by Deinhammer et al. [91] and the existence of covalent carbon-nitrogen bonds was proven by X-ray photoelectron spectroscopy. The technique is not unique to Cys, in fact it has its origins in the deposition of a multitude of amino compounds [92]. Other amino acids, for instance l-glutamate, have since been deposited by the same method [93].

In earlier work, Wang et al. reported correctly that cyclic voltammetry leads to the deposition of l-cysteine [94]. Later on terminology changed and the term p(Cys) was used for the coatings generated by cyclic voltammetry of Cys. Since a complete coating of the electrode is achieved one can assume that the term p(Cys) is now used to signify the formation of a homogenous film that provides the electrode with a distinct macro-porous structure that provides a specific microenvironment, which lends the enhanced sensitivity to electrodes.

Cysteine is unique as it also carries a redox active thiol group. Hence, it needs to be assumed that thiol radicals are formed as well and covalently attach themselves to the active sites of the aromatic carbon moiety in a similar manner as amino radicals do. However, the electron-rich amino group is electro-activated at lower potentials than the thiol group, and the electro-oxidative deposition of thiols usually needs to be facilitated by the presence of strong deprotonating agents [95]. Hence it can be assumed that the deposition of Cys onto electrodes is most likely non-uniform with N–C and S–C covalent bonds forming. Moreover one can expect a variety of non-specific radical side reactions, that eventually lead to the formation of a polymer-resembling network that is pinhole-free and displays uniform properties and is referred to in this context as p(Cys). 

## 5. Conclusions

There is no indication that p(l-Cys) exists as homopolymer in the biological realm. However, the redox activity of the pendant thiol group in Cys repeat units in various peptides and proteins is crucial to protein folding and a potent antioxidant, most notably in the action of glutathione. Poly(cysteine) and p(Cys) containing copolymers have been developed for two very different areas of application: (i) biomedical polymers; and (ii) sensitivity enhancing electrode coatings. In the biomedical realm the crosslinking capacity is exploited to form redox-sensitive nanostructures for drug delivery vehicles. Moreover, the aurophilicity of p(Cys) has been utilized to generate dense polymer coatings on biomedical device surfaces by exploiting the multipoint attachment potential provided by p(Cys) segments in various copolymers. Poly(Cys) containing copolymers for biomedical applications are typically synthesized by well controlled ROPs of the Cys NCA, often copolymerized or block-copolymerized with NCAs of other amino acids. Amino terminated biocompatible polymers lend themselves as macroinitiators and contribute to the amphiphilicity of the resulting construct. The reactivity of the pendant thiol group has been exploited in multiple ways to further derivatize the copolymer. Poly(Cys) for electrochemical applications is very different; it is produced in situ from Cys on electrode surfaces via cyclic voltammetry. The resulting films enhance the electrode sensitivity. However, the two types of p(Cys) are very different; while p(Cys) for biomedical applications is the product of a well-controlled, typically living ROP, the p(Cys) formed by cyclic voltammetry is not truly a polymer and is better described as a deposition of Cys anchored via N-C bonds and a multitude of non-specific side reactions contributes to the formation of dense, pin-hole free coatings. Poly(Cys) is currently in its early stage of academic research for biomedical applications. It can be expected that the redox capacity of the polymer will be the main target to be exploited, as the chemistries of the cytoplasm lend support to pH induced rearrangements of delivery vehicles. The aurophilicity of the polymer contributes another set of exploitable properties and is most likely to be developed for further surface modifications.
